# Modulating the Structure of Motor Variability for Skill Learning Through Specific Muscle Synergies in Elderlies and Young Adults

**DOI:** 10.1109/OJEMB.2019.2963666

**Published:** 2020-02-14

**Authors:** Vincent C. K. Cheung, Xiao-Chang Zheng, Roy T. H. Cheung, Rosa H. M. Chan

**Affiliations:** ^1^ School of Biomedical Sciences and Gerald Choa Neuroscience Centre, and the KIZ-CUHK Joint Laboratory of Bioresources and Molecular Research of Common DiseasesThe Chinese University of Hong Kong 26451 Hong Kong China; ^2^ Department of Rehabilitation SciencesThe Hong Kong Polytechnic University 539133 Hong Kong China; ^3^ Department of Electrical EngineeringCity University of Hong Kong 53025 Hong Kong China

**Keywords:** Ageing, EMG, Motor Learning, Motor Variability, Muscle Synergy

## Abstract

*Objective:* Motor variability – performance variations across task repetitions – has been assumed to be undesirable. But recent studies argue that variability facilitates early motor learning by allowing exploratory search of reward-generating motion, and that variability's structure may be modulated by neural circuits for furthering learning. What are the neural sources of learning-relevant motor variability and its modulation in humans of different ages? *Methods:* Elderlies and young adults played a 3-session virtual bowling while multi-muscle electromyographic signals were collected. We quantified trial-to-trial variability of muscle synergies – neuromotor control modules – and of their activations. *Results:* In elderlies, bowling-score gain correlated with change of activation timing variability of specific synergies, but in young adults, with variability changes of synergy-activation magnitude, and of the synergies themselves. *Conclusions:* Variability modulation of specific muscle synergies and their activations contribute to early motor learning. Elderly and young individuals may rely on different aspects of motor variability to drive learning.

## I. Introduction

Humans do not move robotically. An expert gymnast, after decades of practice on a relatively simple maneuver, would still perform the maneuver a little differently each time. The structure of *motor variability*, defined as “the variation of performance across repetitions or continuous performance of the same task” [1], may be a window for inferring fundamental motor control principles because it is assumed that any neuromotor control strategies must have evolved to minimize the impact of motor variability – an undesirable movement feature – on achieving behavioral goals. In [2], for instance, it was argued that movement planning is based on the selection of a trajectory shape that diminishes the variance of the final end-effector position originating from signal-dependent noise in the motor command.

Recently, multiple investigators have emphasized instead the functional benefits of motor variability in relation to motor learning [4], [31], [43]. As performance improves during learning, motor-output variability eventually decreases [3]. But during early learning, motor variability could allow randomized, active exploration of the motor-command space, thus enabling faster learning because higher variability implies a higher chance of hitting a command conducive to task goal accomplishment. The task-relevant motor command can then be reinforced through rewards for driving learning progress. In a recent study that employed a reward-based reaching task [4], across subjects pre-training kinematic variability along task-relevant dimensions correlated well with subsequent learning rate. Importantly, task training also altered the structure of variability in a way that, after training, motor output varied along the dimensions that complied with the task demands, thus facilitating further learning [4].

The above result implies that motor variability may not result just from random fluctuations (“noise” [5]) of sensorimotor activities, but may be under active regulation during motor learning by dedicated neural circuits. Indeed, during courtship song learning of zebra finches, typically variable songs became highly stereotyped after the lateral magnocellular nucleus (LMAN) was inactivated [6], suggesting that LMAN injects variability into the vocal motor pathway to facilitate song production.

**Figure 1.**
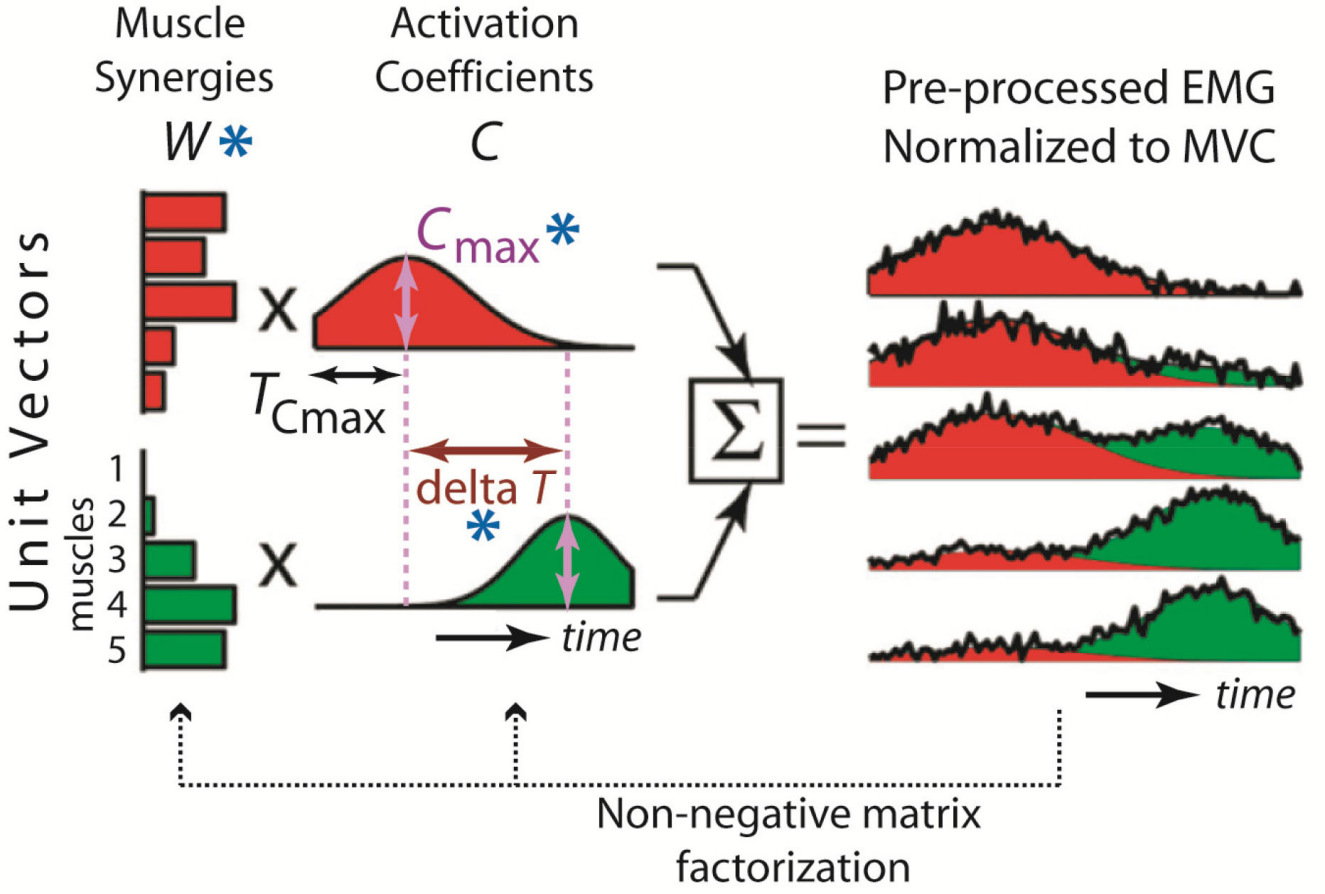
Schematic of muscle synergy combination. Each muscle synergy is a time-invariant unit vector across muscles (*W*), and scaled by a time-varying activation coefficient (*C*). The waveforms from the synergies are linearly summed to explain the EMGs normalized to maximum voluntary contraction (MVC) of the muscles. The quantities whose variability are correlated to performance measures here – *W*, trial maximum of *C* (*C*_max_), and time interval between *C*_max_'s of synergy pairs – are marked by blue asterisks. Figure adapted from [42].

In humans, the neural source of motor variability relevant to learning has remained obscure. Prior studies that explore variability and learning in humans [2], [4] have characterized variability in kinematics or force profile, but not variability in the neuromotor outputs as reflected in neurophysiological recordings. Presumably, motor variability owes most of its origin to variance in the muscle pattern, which in turn arises in part from fluctuations of the neuronal activities that coordinate muscle activations. One neuronal type whose activity fluctuation should result in myoelectric variability is the one that encodes *muscle synergies* – neuromotor modules that coordinate muscle activities with across-muscle activation balance profiles scaled by time-varying coefficients, and whose combination could explain the experimental electromyographic signals (EMGs) (Fig. 1) [7]–[10]. Muscle synergies are popularly identified by applying factorization algorithms to multi-muscle EMGs [11], [12]. Recent neurophysiological data have argued that factorization-derived muscle synergies may reflect muscle coordinative structures encoded by lower-level spinal interneuronal networks whose temporal activations are specified by the afferent systems and descending drives from the higher-level motor areas [10], [13]–[17].

Given that muscle synergies may be entities of neural origin [18], [19] and that variations of multi-muscle activities may promote early motor learning, it is possible that trial-to-trial variability of the muscle synergies themselves and/or their activation patterns could be meaningful fluctuations that drive learning. Here, we ask whether learning-relevant motor variability owes its origin to (1) fluctuations of the higher-level neural commands that drive the downstream muscle synergies, or (2) activation variability of the lower-level synergy-encoding interneurons and motoneurons that result in fluctuation of the muscle synergies themselves. We addressed this question by examining whether trial-to-trial variability of muscle-synergy activations, or variability of the muscle synergies, correlate with the initial learning rate in the early phase of a reward-based task. This should allow a better understanding of the neural sources of learning-relevant variability in humans.

In addition, we seek to reveal here the difference between young and old individuals in how motor learning is achieved *vis-à-vis* their reliance on motor variability in driving learning. Per previous results, elderlies, when compared with young adults, have a more-or-less preserved potential to acquire motor skills. Some studies have failed to detect any difference in the rate and extent of learning between younger and older groups [20], [21]. Others have found that elderlies could reach almost the same degrees of skill learning as those of young adults, albeit with slower learning rates [22]–[25], in tasks including mirror tracking [22] and rotary pursuit task [22]. We therefore hypothesized that young adults can better exploit and modulate motor variability for achieving their motor-learning goals with faster rates than elderlies, and anticipated a salient correlation between muscle-synergy variability and learning rate in young adults but not elderlies.

## II. Results

**Figure 2.**
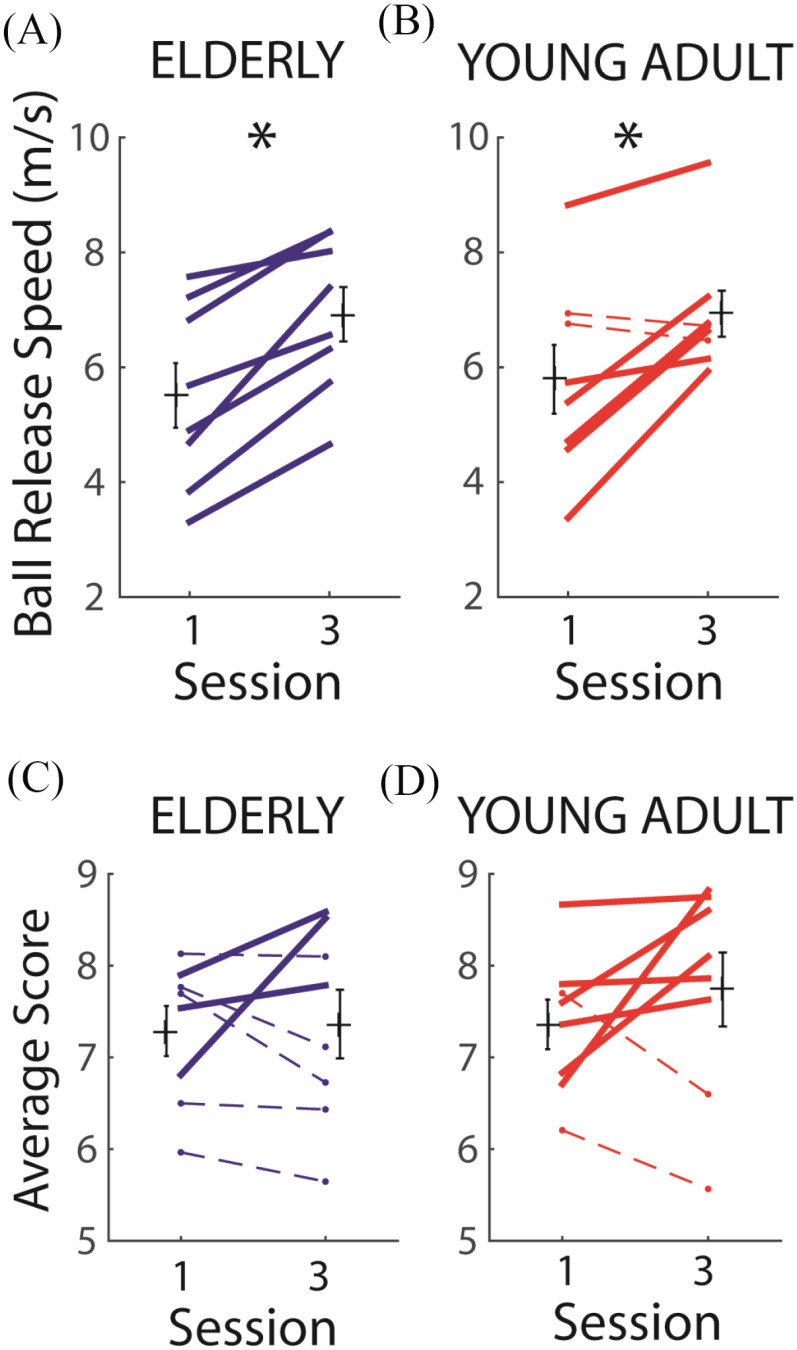
Measures for assessing game performance across sessions. *A*,*B*, The release speed of the virtual ball was estimated by calculating the peak forward speed of a reflective marker on the wrist (Vicon). In both elderlies (*A*) and young adults (*B*), there was an increase of ball release speed (group mean ± SE) from session 1 to 3 (t-test, p < 0.05, *). Thick lines, subjects showing an increase; dotted lines, subjects showing a decrease. *C,D,* We also monitored the number of pins knocked down in the first throw of each trial (bowling score), averaged across all trials of a session. In both groups, differences between session mean scores were not statistically significant (t-test, p > 0.05). But there were more young adults (*D*) showing score increases (6 of 8, thick lines) then elderlies (*C*) (3 of 8). Note the substantial across-subject variability in score increases in both groups. Note also that only the score achieved, but *not* ball release speed, was explicitly fed back to each subject during the game.

Young adults (N = 8, mean age = 23.8) and elderlies (N = 8, mean age = 71.1) were trained to play a virtual bowling game for 3 daily sessions. To make scoring more difficult, all subjects were trained to bowl with the non-dominant arm. Game performance was quantified by the ball release speed (derived from wrist kinematics) and the bowling score (average number of knocked-down pins per trial) recorded on the first and last sessions. Both age groups exhibited equivalent increase in ball release speed from sessions 1 to 3 (p < 0.01; Fig. 2(A)–(B)), but showed no statistically significant change in the across-subject average of the bowling score (p > 0.05; Fig. 2(C)–(D)). We note, however, that in both groups there was substantial across-subject variability in how the bowling score changed. Also, while 6 of 8 young adults showed an increase in bowling score (Fig. 2(D), thick red), only 2 of 8 elderlies showed an increase (Fig. 2(C), thick blue). Thus, both age groups were capable of kinematic change after training, but more young adults were able to derive reward (i.e., scores) from aspects of kinematic change including, but not limited to, the ball release speed.

To address our scientific question of whether there is any age-related difference in how the muscle synergies are changed by bowling training, we began by performing a “conventional” muscle synergy analysis on EMGs (11 muscles) that were normalized to levels at maximum voluntary contraction of the muscles. We identified muscle synergies from the EMGs of whole sessions using non-negative matrix factorization (NMF) [26], [27] (Fig. 1). The extracted synergies were then compared between sessions and groups, with similarity between synergy sets quantified by the average scalar product between matched muscle-synergy pairs [10]. This analysis did not reveal any salient age-related difference in muscle synergy during learning (Fig. S1-S4).

The above result notwithstanding, it is possible that insights into age-related difference in the muscle synergies (*W*) and/or their activations (*C*) during learning can only be revealed by going beyond a straight-forward inter-group comparison of the *W*'s. Below, we will characterize, in two analytic stages, the trial-to-trial variability of *W* and *C* of each session of every subject, and asked whether the initial *W*- or *C-*variability in session 1 correlates with the change of ball release speed or bowling score from session 1 to 3 in either age group. We also went one step further and examined whether the across-session *change* of *W-* and *C*-variability correlates with the change of the performance measures, assuming that *W-* and *C*-variability are themselves modulated by the nervous system [4], [6] for driving motor behavioral change.

### A. Change of Variability of Synergy Activation Magnitude Correlated With Bowling Score Change in Young Adults But Not Elderlies

In the first stage of our variability analysis, for every subject we characterized the spatiotemporal variability of *C* with a *W* that was fixed through all trials of each session. To ensure correspondence of the *W*'s for the two sessions so that the change of *C* variability could be meaningfully quantified, the *W* of either session was extracted by initializing NMF with the same initial guess, one that was the cluster centroids from *k*-means clustering (9 clusters, Fig. S4) of the *W*'s identified from the combined EMGs of sessions 1 and 3 of all subjects in each age group. This way, NMF was provided with prior knowledge of *W* based on its average representation across all sessions of the group. The *C*-magnitude variability of a synergy was quantified by the across-trial variance of its trial-maximum magnitude (*C*_max_). Both the mean and maximum, across the set of synergies of each subject, of the initial and change of *C*_max_ variability were calculated as predictors of the change in performance measures, thus making 4 (predictors) × 2 (performance measures) = 8 correlations to consider in each group.

**Figure 3.**
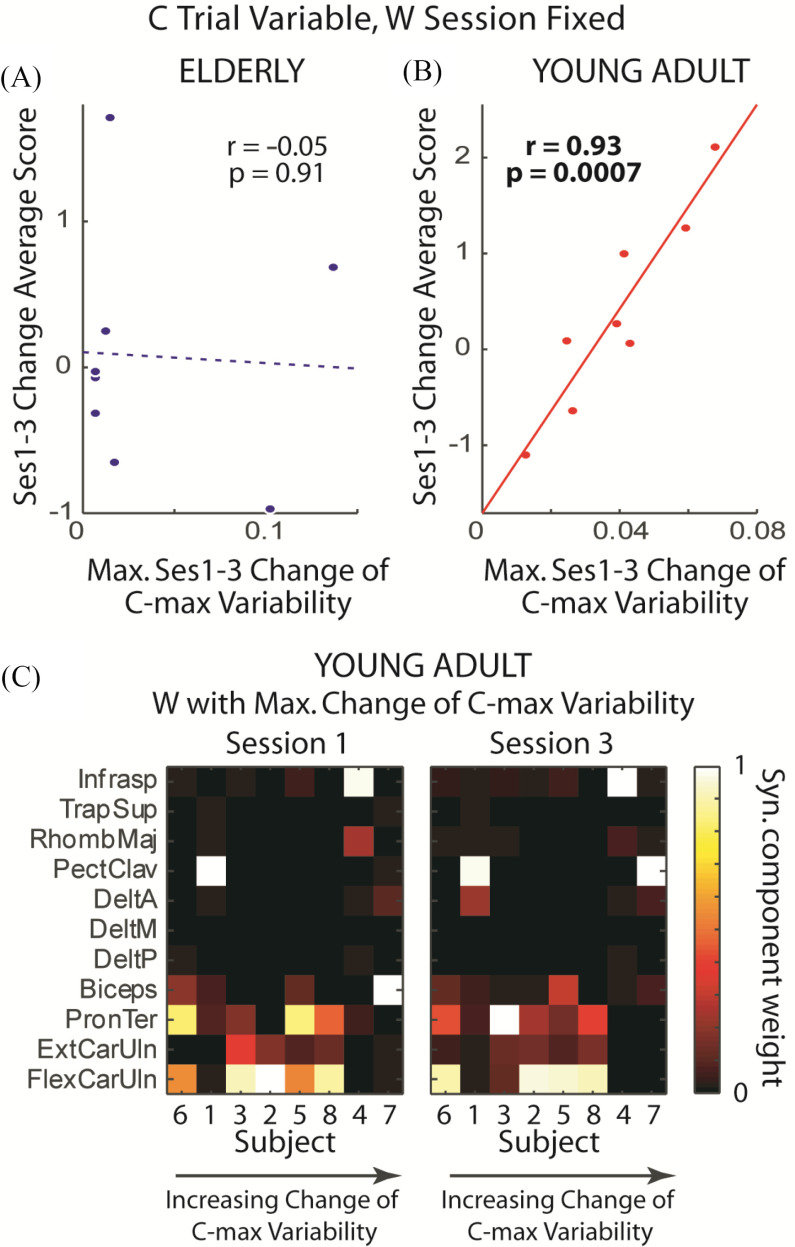
Characterization of *C*-magnitude variability when *W* was fixed across the trials of a session. *A,B,* In both age groups, the change of average bowling score was correlated against the maximum (across each subject's synergies) session-1-to-3 change of *C*_max_ variability. A highly statistically significant correlation was found in young adults (*B*, solid line) but not elderlies (*A*, dotted line). r, Pearson's correlation coefficient; p, the p-value of the correlation. *C*, The muscle synergies (*W*) showing maximum *C*_max_ variability in the young adults (each row corresponds to a muscle; each column, a subject). Note consistency of these session-fixed *W*'s from session 1 to 3. Also note how muscles including Biceps, PronTer, and FlexCarUln were consistently recruited. Interestingly, their biomechanical functions – wrist/elbow flexion and wrist internal rotation – align with those demanded of high-scoring bowlers [39].

Among the correlations involving the bowling score as the response variable, we found that the maximum change (from session 1 to 3) of *C*_max_ variability correlated positively, and very significantly, with the change of bowling score in young adults (Pearson's *r* = 0.93, p = 0.0007) but not elderlies (p = 0.91) (Fig. 3(A)–(B)). We verified that across young adults, the *W*'s corresponding to the *C*'s with maximum *C*_max_-variability changes remained almost unaltered from session 1 to 3 (Fig. 3(C)). Interestingly, for 5 of 8 subjects, these *W*'s involved the same set of elbow and wrist flexors relevant to ball release – flexor carpi ulnaris (FlexCarUln), pronator teres (PronTer), and biceps brachii (Biceps) (Fig. 3(C)).

It is true that with 9 synergies (from 11 muscles), about half of all session-fixed *W*'s were dominated by single muscles (% of synergies with just 1 muscle with >0.1 component weight: elderlies session 1, 40%, session 3, 40%; young adults session 1, 46%, session 3, 44%;). But the value of muscle synergy analysis can be readily appreciated by noting the following: (1) correlation between score change and maximum EMG_max_-magnitude variability change of individual muscles in young adults yielded a much lower *r* with borderline statistical significance (*r* = 0.72, p = 0.045); (2) most synergies showing maximum *C*_max_-variability change in young adults involved >1 muscles (Fig. 3(C)). In fact, when the same analysis was performed by initializing NMF at the original, lower dimensionality of each subject (elderlies, number of synergies = 5.6 ± 0.7 at R^2^∼90%; young adults, 6.3 ± 0.9), we still obtained a very significant correlation *only* in young adults between score change and maximum *C*_max_-magnitude variability change (elderlies, p = 0.87; young adults, *r* = 0.92, p = 0.001).

In addition, we found a significant, but weaker, negative correlation between the mean initial *C*_max_ variability and change of ball release speed in elderlies (*r* = −0.74, p = 0.03) but not young adults (p = 0.69). No other significant correlation involving *C*_max_ magnitude was observed in this stage (Table S1 lists all *r* and p values).

### B. Initial and Change of Variability of Synergy Activation Timing Correlated With Bowling Score Change in Elderlies But Not Young Adults

**Figure 4.**
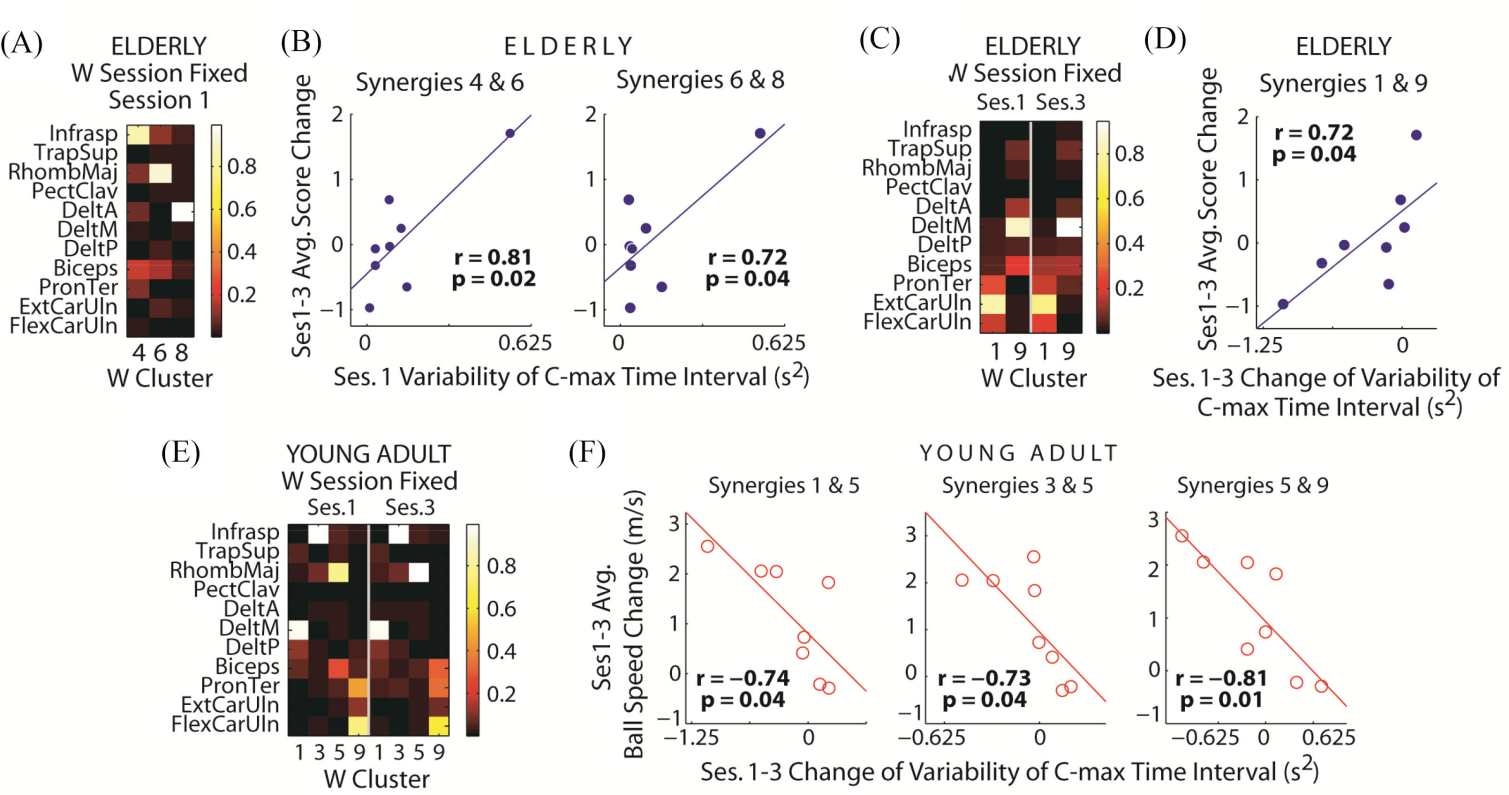
Characterization of *C*-timing variability when *W* was fixed across the trials of a session. Variability of *C* timing was quantified by the variance of time interval between the *C*_max_'s of every synergy pair. *A,B,* In elderlies, score gain correlated significantly with the initial (session 1) time-interval variability of 2 synergy pairs (*B*) involving 3 different *W*'s (*A*). *C, D,* In elderlies, score gain also correlated significantly with the change (from session 1 to 3) of time-interval variability of 1 synergy pair (*D*) involving 2 *W*'s (*C*). Again, note consistency of the *W*'s from session 1 to 3. Interestingly, the pair of *W*'s corresponding to the *C*'s in panel C included activation components in the same set of elbow/wrist flexors we noted earlier (FlexCarUln, PronTer, Biceps) in Fig. 3(C) as well as medial deltoid (DeltM). *E, F,* In young adults, time-interval variability correlated significantly, and negatively, only with the change of ball release speed (*F*) but not score gain. Three significant correlations involving 4 *W*'s (*E*) were identified.

We proceeded to characterize the initial and change of the variability of *C* activation timing, defined first by noting, in every synergy and trial, the time at which *C*_max_ occurred (*T*_Cmax_), and then calculating the across-trial variance of the time interval between the *T*_Cmax_'s of every pair of the 9 synergies (Fig. 1). When the bowling score change was the response variable, we found only 3 statistically significant correlations, all positive and observed only in elderlies but not young adults; 2 of 3 involved the initial (session 1) time-interval variability as covariates (Fig. 4(A)–(B)), and 1 involved the change (from session 1 to 3) of time-interval variability (Fig. 4(C)–(D)).

When the change of ball release speed was the response variable, we identified 3 negative correlations in young adults – all with the change of variability as covariates – that were statistically significant (Fig. 4(E)–(F)). In elderlies, the initial *T*_Cmax_-time interval between two synergies, dominated by pectoralis major and infraspinatus, respectively, also correlated significantly with the change of ball speed (*r* = 0.78, p = 0.02) (not shown). Beyond these correlations, none of the others in either group was significant.

### C. Change of Muscle-Synergy Variability Correlated With Bowling Score Change in Young Adults But Not Elderlies

The variability analysis described above aimed at understanding the variability of *C* while assuming a session-fixed *W*. In our second stage of analysis, we entertained the possibility that *W* may itself exhibits trial-to-trial variability that is relevant to learning. Such an approach would demand a new computational formulation that can reasonably estimate trial-specific *W*'s from the EMGs – and importantly, while having the across-trial averages of the trial-specific *W*'s to still *globally* explain the data – so that a trial-specific *W* would represent a variant of a “true global *W*” rather than just a fit to the data peculiarities of the trial. To achieve this, we developed a procedure that exploits the fact that in NMF, the initial estimate of *W* corresponds to prior knowledge of how *W* may be structured [27], [28]. It follows that a trial-specific *W*, as understood above, can be identified by initializing NMF with the “global” *W* extracted from the EMGs of all trials, so that the extraction of the trial-specific *W* amounts to fine-tuning the global *W* to suit the EMGs of each trial. This approach is conceptually similar to how an artificial neural network can be pretrained to learn general-purpose features by another data set before being fine-tuned for the target task [29], [30].

**Figure 5.**
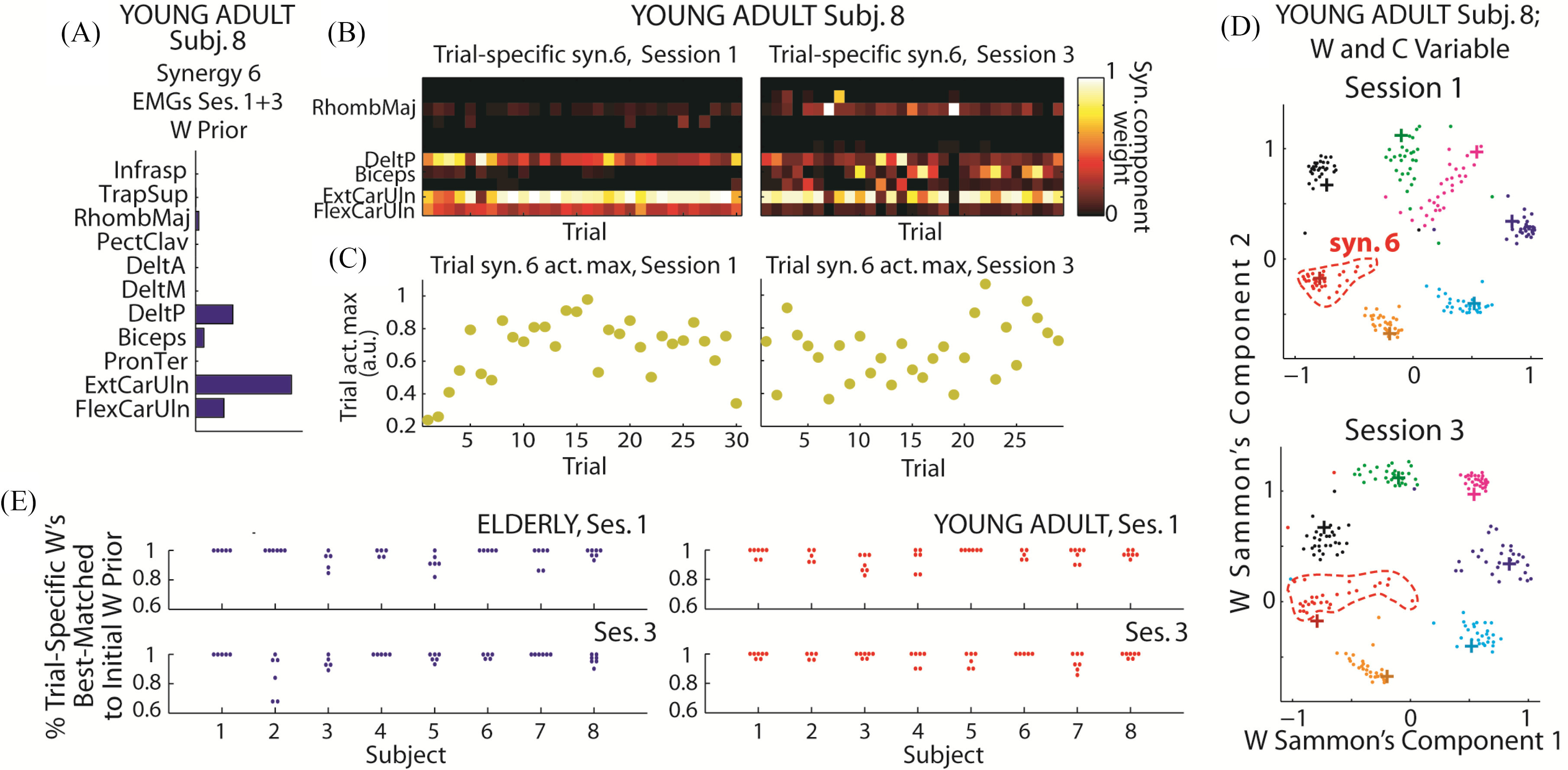
Modeling motor variability with trial-variable *W* and *C*. *A*, The initial guess for *W* in the NMF corresponds to prior information known about *W*; thus, the global estimate of *W* (extracted from the combined EMGs of sessions 1 and 3) could be supplied to NMF as “W prior” for extracting trial-specific *W*'s. *B*, The trial-specific *W*'s from the prior shown in *A*. Each column corresponds to the *W* of a trial. *C*, The *C*_max_ resulting from the extraction of trial-specific *W*'s, for the same trials shown in *B*. Note that *C*_max_-variability remained even when *W* was allowed to be trial-variable. *D*, We visualized the trial-specific *W*'s (.) and their *W* priors (+) by projecting all *W* vectors to 2-D space by Sammon mapping. Each color denotes an individual synergy. Note that the *W* priors used in both sessions were the same. Synergy 6 here (red) corresponds to the synergy shown in *A,* and *B*. Note the increase of variance of synergy 6. *E*, To validate the consistency of the trial-specific *W*'s resulting from each *W* prior, we matched every set of trial-specific *W*'s with the set of *W* priors by maximum scalar product, and for each prior, calculated the percentage of trials with its trial-specific *W*'s matched back to itself. Each data point on the graph denotes the percentage of a synergy. Across subjects, the percentages were consistently high (∼90–100%).

In Fig. 5 we demonstrated the extraction of trial-specific *W*'s with an example of the initial guess for *W* that was identified from the combined EMGs of sessions 1 and 3 (Fig. 5(A)), and all the trials-specific *W*'s and *C*_max_'s that resulted from this prior (Fig. 5(B)–(C)). We validated that the same prior would not lead to the identification of grossly different synergies across trials by matching each set of trial-specific *W* to the set of *W* prior, and showing that most of the trial-specific synergies indeed were matched back to their own priors (Fig. 5(E)). The variability of the *W*'s can also be appreciated in Fig. 5(D) in which we projected the *W* prior (+) and all trial-specific *W*'s (.) of a subject onto a 2-D plane by Sammon mapping. Specifically, the *W* variability of a synergy (red) increased conspicuously from session 1 to 3 while those of others decreased or remained unchanged.

**Figure 6.**
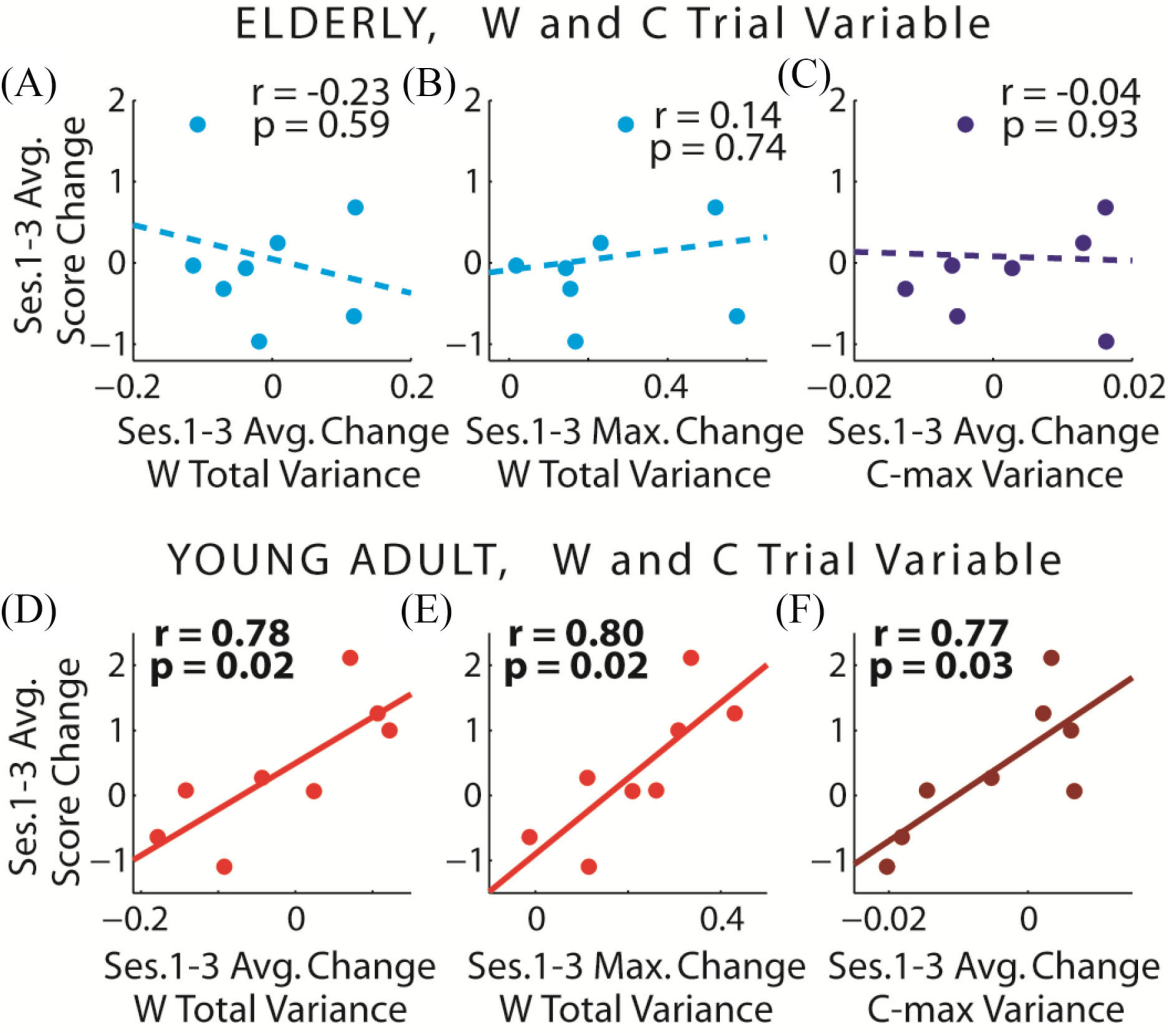
Correlating *W* and *C* variability with performance measures. *A-C*, In elderlies, we did not find any statistically significant correlations between bowling score gain and the change (from session 1 to 3) of *W* and *C* variability. *D-F,* In young adults, on the other hand, the mean (across the synergies) (*D*) and maximum (*E*) change of *W* variability, and the mean change of *C*_max_ variability (*F*) correlated significantly with score gain.

We proceeded to quantify *W* variability of each synergy by calculating its total across-trial variance, and correlate this variability against change of performance measures. We found that both the mean and maximum (across the set of synergies) change of *W* variability correlated positively and significantly with the change of bowling score in young adults (Fig. 6(D)–(E)) but not elderlies (Fig. 6(A)–(B)). In addition, we found a significant correlation between the mean change of *C*_max_-magnitude variability with the change of bowling score, again only in young adults (Fig. 6(F)) but not elderlies (Fig. 6(C)), consistent with the result obtained when *W* was session-fixed (Fig. 3(B)). Otherwise, none of the other correlations were found to be significant (Table S2-S3).

## III. Discussion

**Figure 7.**
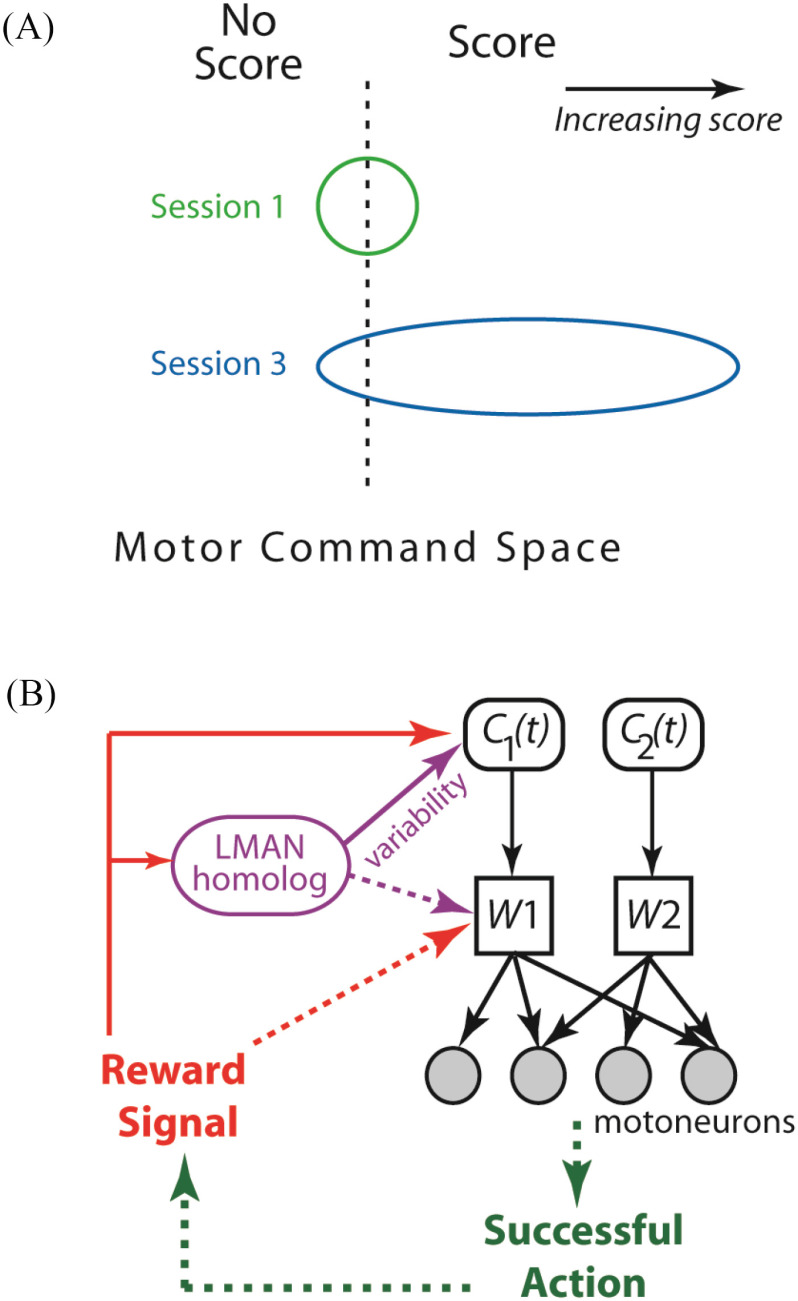
Modulating the structure of motor variability through specific muscle synergies. *A*, A schematic illustrating how increasing the variability of a specific *W* or its *C* from session 1 (green circle) to session 3 (blue ellipse) could result in score gain. Score gain can be facilitated if *W* or *C* variability is modulated such that the subspace of motor commands from *W* and *C* is enlarged towards the command subspace that is conducive to scoring. *B,* A hypothetical neural circuit for modulating learning-relevant motor variability. The conjectured human homolog of the finches’ LMAN injects variability to the higher motor areas for generating the *C* for a *specific* synergy (solid purple line), and for young adults only, to the lower-level brainstem/spinal circuits for structuring that synergy, *W* (dotted purple line). Reward signals from successful actions serve to reinforce the *W* and *C* variants conducive to task achievement, and also the LMAN for that synergy, so that the synergy's variability can be modulated for more reward-generating actions.

The most salient result here is that in both age groups, excepting the 2 correlations involving the initial *C*-timing variability of elderlies, by and large the *change* (rather than the initial) of variability of either the muscle synergies (*W*) or the magnitude or timing of their activations (*C*) correlated with the gain of bowling score. For elderlies, variability change of *C* activation time was relevant to score gain (Fig. 4(D)); for young adults, variability changes of both *W* (Fig. 6(D)–(E)) and *C* activation magnitude (Fig. 3(B); Fig. 6(F)) were relevant. These results suggest that variability of *specific* task-relevant muscle synergies and/or their spatiotemporal activation patterns may be suitably modulated by the CNS during early learning, so that the structure of motor-command variability within the space of commands would be biased towards the directions conducive to furthering task-goal achievement (Fig. 7(A)). This interpretation is consistent with the finding of Wu *et al.* [4], that motor training altered the kinematic variability structure so that post-training motor output varied along the task-compliant dimensions, thus facilitating more learning. Our results further suggest that this observation in [4] may be underpinned by variability modulation of select muscle synergies and/or their activations.

We speculate that variability modulation of specific *W*'s and *C*'s may be underscored by a neuronal network functionally similar to the zebra finch lateral magnocellular nucleus (LMAN), which injects variability into the vocal motor pathway during early song learning [6], [31]. The human homolog of LMAN has not been identified [32], but conceivably it could be a network within the various premotor areas [33]. To modulate the synergies’ variability, this conjectured LMAN homolog should have access to the networks for the *W*'s and *C*'s, and be susceptible to modulation by the reward signals that reinforce any specific *W* and *C* variants resulting in reward-generating actions. The reward reinforcement would then direct how LMAN should modulate the variability levels of different synergies, thereby altering the structure of motor-command variability in a manner that favors rewarding outcomes (Fig. 7(B)).

Another salient result from our analysis concerns how elderlies relied on the initial and change of *C*-timing variability for score gain (Fig. 4(A)–(D)) while young adults relied on the change of *C*-magnitude variability (Fig. 3(B), Fig. 6(F)). Elderlies, when compared with young adults, have less muscle strength [34], [35], and hence are less able to produce and sustain high-magnitude muscle forces. Because of the decreased maximal force, the elderly motor system is probably less capable of generating functionally sufficient motor variability by varying muscle-activation magnitude, and has therefore learned to depend instead on timing variability for driving learning. We speculate that for many motor tasks, varying the timing of *C* may be a less effective strategy for learning than one based on varying magnitude, and this may partially explain why elderlies display slower learning rates than young adults in prior studies [22]–[25]. We note, however, that elderlies’ slower learning may also be attributed to their striatal and dopaminergic systems whose activations are less sensitive to the changing reward outcomes [36], [37].

Our study is limited in several ways. We have characterized motor variability and performance during early learning by training our subjects for 3 sessions, but we do not know whether the initial or change of variability during these first sessions would predict the learning rate in the ensuing sessions during longer-term training. Answering this question would require an even more demanding motor task. Even though bowling is biomechanically fairly complex [38]–[41], by session 3 both age groups already managed to achieve ∼7–8/10 pins per trial (Fig. 2(C)–(D)). A more challenging task would permit long-term behavioral changes to be manifested.

## IV. Conclusions

We have demonstrated that trial-to-trial fluctuations of specific muscle synergies and their spatiotemporal activations can be sources of learning-relevant motor variability. Our results imply that one strategy to accelerate motor learning – especially when the “best” muscle patterns for the task is not known *a priori* – may be to promote and modulate the variability of specific synergies or their activations. Our analyses have also defined a paradigm for characterizing variability of multi-muscle coordination, which could be useful in many applications in neural and rehabilitation engineering.

## Supplementary Materials

The Results section of supplementary materials contains a detailed description of the conventional muscle synergy analysis performed on the EMGs (Fig. S1-S4). Supplementary materials also includes a full description of the Materials and Methods employed for this study. Finally, the tables in Supplementary Materials (Table S1-S3) list all Pearson's *r* correlation coefficients and their associated p values for the correlations performed in both stages of our *W* and *C* variability analyses.



## References

[1] K. He, “The statistical determinants of the speed of motor learning,” PLoS Compt. Biol., vol. 12, no. 9, Sep. 2016, Art. no. e1005023. 10.1371/journal.pcbi.1005023 PMC5015831 27606808

[2] C. M. Harris and D. M. Wolpert, “Signal-dependent noise determines motor planning,” Nature, vol. 394, no. 6695, p. 780, Aug. 1998. 9723616 10.1038/29528

[3] H. Müller and D. Sternad, “Motor learning: Changes in the structure of variability in a redundant task,” in Progress in Motor Control 2009, Boston, MA: Springer, 2009, pp. 439–456. 10.1007/978-0-387-77064-2_23 PMC3776417 19227514

[4] H. G. Wu , “Temporal structure of motor variability is dynamically regulated and predicts motor learning ability,” Nat. Neurosci., vol. 17 no. 2, pp. 312–321, Feb. 2014. 24413700 10.1038/nn.3616 PMC4442489

[5] A. A. Faisal , “Noise in the nervous system,” Nature Rev. Neurosci., vol. 9, no. 4, p. 292, Apr. 2008. 18319728 10.1038/nrn2258 PMC2631351

[6] B. P. Ölveczky , “Vocal experimentation in the juvenile songbird requires a basal ganglia circuit,” PLoS Biol., vol. 3, no. 5, Mar. 2005, Art. no. e153. 10.1371/journal.pbio.0030153 PMC1069649 15826219

[7] M. C. Tresch , “The construction of movement by the spinal cord,” Nat. Neurosci., vol. 2, no. 2, p. 162, Feb. 1999. 10195201 10.1038/5721

[8] P. Saltiel , “Muscle synergies encoded within the spinal cord: Evidence from focal intraspinal NMDA iontophoresis in the frog,” J. Neurophysiol., vol. 85, no. 2, pp. 605–19, Feb. 2001. 11160497 10.1152/jn.2001.85.2.605

[9] A. d'Avella and E. Bizzi, “Shared and specific muscle synergies in natural motor behaviors,” PNAS, vol. 102, no. 8, pp. 3076–81, Feb. 2005. 15708969 10.1073/pnas.0500199102 PMC549495

[10] V. C. K. Cheung , “Central and sensory contributions to the activation and organization of muscle synergies during natural motor behaviors,” J. Neurosci., vol. 25, no. 27, pp. 6419–34, Jul. 2005. 16000633 10.1523/JNEUROSCI.4904-04.2005 PMC6725265

[11] M. C. Tresch , “Matrix factorization algorithms for the identification of muscle synergies: Evaluation on simulated and experimental data sets,” J. Neurophysiol., vol. 95, no. 4, pp. 2199–212, Apr. 2006. 16394079 10.1152/jn.00222.2005

[12] K. Devarajan and V. C. K. Cheung, “A quasi-likelihood approach to nonnegative matrix factorization,” Neural Compt., vol. 28, no. 8, pp. 1663–93, Aug. 2016. 10.1162/NECO_a_00853 PMC5549860 27348511

[13] S. A. Overduin , “Microstimulation activates a handful of muscle synergies,” Neuron, vol. 76, no. 6, pp. 71–7, Dec. 2012. 10.1016/j.neuron.2012.10.018 PMC3547640 23259944

[14] S. L. Amundsen Huffmaster , “Muscle synergies obtained from comprehensive mapping of the primary motor cortex forelimb representation using high-frequency, long-duration ICMS,” J. Neurophysiol., vol. 118, no. 1, pp. 455–70, Apr. 2017. 28446586 10.1152/jn.00784.2016 PMC5506266

[15] T. Takei , “Neural basis for hand muscle synergies in the primate spinal cord,” PNAS, vol. 114, no. 32, pp. 8643–8, Aug. 2017. 28739958 10.1073/pnas.1704328114 PMC5559022

[16] E. Desrochers , “Spinal control of muscle synergies for adult mammalian locomotion,” J. Physiol., vol. 597, no. 1, pp. 333–50, Jan. 2019. 30334575 10.1113/JP277018 PMC6312424

[17] Q. Yang , “Motor primitives are determined in early development and are then robustly conserved into adulthood,” PNAS, vol. 116, no. 24, pp. 12025–34, Jun. 2019. 31138689 10.1073/pnas.1821455116 PMC6575561

[18] E. Bizzi and V. C. K. Cheung, “The neural origin of muscle synergies,” Front. Compt. Neurosci. vol. 7, Apr. 2013, Art. no. 51. 10.3389/fncom.2013.00051 PMC3638124 23641212

[19] V. C. K. Cheung, C. M. Niu, S. Li, Q. Xie, and N. Lan, “A novel FES strategy for poststroke rehabilitation based on the natural organization of neuromuscular control,” IEEE Rev. Biomed. Engin., vol. 12, pp. 154–167, Oct. 2018. 10.1109/RBME.2018.2874132 30307876

[20] V. Gaillard , “Effects of age and practice in sequence learning: A graded account of ageing, learning, and control,” Eur. J. Cog. Psych., vol. 21, no. 2-3, pp. 255–82, Mar. 2009.

[21] G. Chauvel , “Age effects shrink when motor learning is predominantly supported by nondeclarative, automatic memory processes: Evidence from golf putting,” Quart. J. Exp. Psych., vol. 65, no. 1, pp. 25–38, Jan. 2012. 10.1080/17470218.2011.588714 21736434

[22] B. M. Wright and R. B. Payne, “Effects of aging on sex differences in psychomotor reminiscence and tracking proficiency,” J. Gerontology, vol. 40, no. 2, pp. 179–84, Mar. 1985. 10.1093/geronj/40.2.179 3973358

[23] S. M. Daselaar , “Similar network activated by young and old adults during the acquisition of a motor sequence,” Neurobiol. Aging, vol. 24, no. 7, pp. 1013–9, Nov. 2003. 12928061 10.1016/s0197-4580(03)00030-7

[24] T. Wu and M. Hallett, “The influence of normal human ageing on automatic movements,” J. Physiol., vol. 562, no. 2, pp. 605–15, Jan. 2005. 15513939 10.1113/jphysiol.2004.076042 PMC1665504

[25] C. H. Lin , “Age related differences in the neural substrates of motor sequence learning after interleaved and repetitive practice,” NeuroImage, vol. 62, no. 3, pp. 2007–20, Sep. 2012. 22584226 10.1016/j.neuroimage.2012.05.015

[26] D. D. Lee and H. S. Seung, “Learning the parts of objects by non-negative matrix factorization,” Nature, vol. 401, no. 6755, pp. 788–791, Oct. 1999. 10548103 10.1038/44565

[27] K. Devarajan and V. C. K. Cheung, “On nonnegative matrix factorization algorithms for signal-dependent noise with application to electromyography data,” Neural Compt., vol. 26, no. 6, pp. 1128–68, Jun. 2014. 10.1162/NECO_a_00576 PMC5548326 24684448

[28] V. C. K. Cheung and M. C. Tresch, “Non-negative matrix factorization algorithms modeling noise distributions within the exponential family,” in Proc. 2005 IEEE Eng. Medicine Biol. 27th Annu. Conf., Jan. 2006, pp. 4990–4993. 10.1109/IEMBS.2005.1615595 17281365

[29] M. Huh , “What makes ImageNet good for transfer learning?” Comput. Vision Pattern Recognit., Aug. 2016, *arXiv:1608.08614*.

[30] W. Pan , “Transfer learning to predict missing ratings via heterogeneous user feedbacks,” in Proc. 22nd Int. Joint Conf. Artif. Intell., Jun. 2011.

[31] A. K. Dhawale , “The role of variability in motor learning,” Ann. Rev. Neurosci., vol. 40, pp. 479–98, Jul. 2017. 28489490 10.1146/annurev-neuro-072116-031548 PMC6091866

[32] A. R. Pfenning , “Convergent transcriptional specializations in the brains of humans and song-learning birds,” Science, vol. 346, no. 6215, Dec. 2014, Art. no. 1256846. 25504733 10.1126/science.1256846 PMC4385736

[33] E. D. Jarvis, “Learned birdsong and the neurobiology of human language,” Annals New York Acad. Sci., vol. 1016, pp. 749–777, Jun. 2004. 10.1196/annals.1298.038 PMC2485240 15313804

[34] D. A. Skelton , “Strength, power and related functional ability of healthy people aged 65–89 years,” Age Ageing, vol. 23, no. 5, pp. 371–7, Sep. 1994. 7825481 10.1093/ageing/23.5.371

[35] V. A. Hughes , “Longitudinal muscle strength changes in older adults: Influence of muscle mass, physical activity, and health,” J. Gerontology Series A: Biol. Sci. Med. Sci., vol. 56, no. 5, pp. B209–17, May 2001. 10.1093/gerona/56.5.b209 11320101

[36] S. Karlsson , “Modulation of striatal dopamine D1 binding by cognitive processing,” NeuroImage, vol. 48, no. 2, pp. 398–404, Nov. 2009. 19539768 10.1016/j.neuroimage.2009.06.030

[37] L. Bäckman , “Linking cognitive aging to alterations in dopamine neurotransmitter functioning: Recent data and future avenues,” Neurosci. Biobehav. Rev., vol. 34, no. 5, pp. 670–7, Apr. 2010. 20026186 10.1016/j.neubiorev.2009.12.008

[38] K. Kimura , “Measurement and analysis of bowling swing motion using 3D acceleration and gyro sensors,” in Proc. IEEE SICE Annual Conference 2011, Sep. 2011, pp. 1115–1119.

[39] R. Razman , “Anthropometric and strength characteristics of tenpin bowlers with different playing abilities,” Biol. Sport, vol. 29, no. 1, pp. 33–38, Mar. 2012.

[40] R. Razman , “Front foot slide variability and its relation to tenpin bowling performance,” in Proc. ISBS-Conf. Archive, 2010, vol. 1, no. 1.

[41] P. D. Hurrion , “Simultaneous measurement of back and front foot ground reaction forces during the same delivery stride of the fast-medium bowler,” J. Sports Sci., vol. 18, no. 12, pp. 993–7, Jan. 2000. 11138989 10.1080/026404100446793

[42] V. C. K. Cheung , “Muscle synergy patterns as physiological markers of motor cortical damage,” PNAS, vol. 109, no. 36, pp. 14652–6, Sep. 2012. 22908288 10.1073/pnas.1212056109 PMC3437897

[43] S. Levy-Tzedek, “Motor errors lead to enhanced performance in older adults,” Sci. Rep., vol. 7, Jun. 2017, Art. no. 3270. 10.1038/s41598-017-03430-4 PMC5468294 28607449

