# Supplementary material for: Modulating the Structure of Motor Variability for Skill Learning Through Specific Muscle Synergies in Elderlies and Young Adults
Source: IEEE Open J Eng Med Biol. 2020 Feb 14;1:33–40. doi: 10.1109/OJEMB.2019.2963666 (PMC8979619; doi:10.1109/OJEMB.2019.2963666)
Supplement: Supplementary file 1 [file supp1-2963666.pdf]

Supplementary Materials

Modulating the Structure of Motor Variability for Skill Learning through Specific Muscle Synergies in Elderlies and Young Adults

Vincent C. K. Cheung, *Member*, *IEEE*, Xiao-Chang Zheng, Roy T. H. Cheung, Rosa H. M. Chan, *Senior Member*, *IEEE*

I. Results

A. Conventional muscle synergy analysis did not reveal any salient age-related difference in muscle synergy during learning

As mentioned in the main text, as a first step to reveal any potential age-related difference in how the muscle synergies are changed by bowling training, we performed a "conventional" muscle synergy analysis on preprocessed electromyographic data (EMGs; 11 muscles) that were normalized to the levels at maximum voluntary contraction of the muscles. We identified muscle synergies from the EMGs of whole sessions using the standard NMF algorithm [1,2]. The extracted synergies were then compared between sessions and groups, with similarity between synergy sets quantified by the average scalar product between matched muscle-synergy pairs [3].

In both age groups and both sessions, the EMGs could be

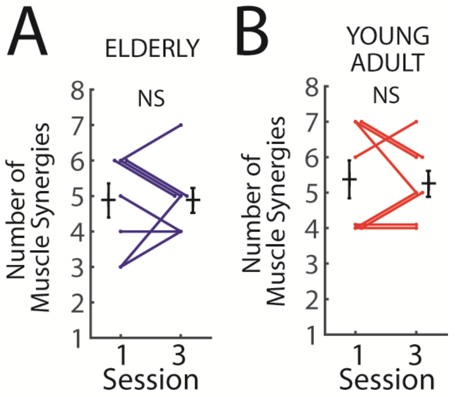

Fig. S1. Dimensionality of the EMGs from elderlies (*A*) and young adults (*B*). No statistically significant change (NS) in dimensionality from session 1 to 3 was detected in either group (t-test, p > 0.05; group mean ± SE).

explained at $R^2 \approx 90\%$ by ~5 muscle synergies, with no statistically significant inter-group or -session differences in this dimensionality (p>0.05; Fig. S1). Across age groups, the scalar-product similarity between every young adult-elderly subject pair did not significantly differ between sessions 1 and 3 (p>0.05; Fig. S2A). Longitudinally, from session 1 to 3, both age groups showed the same degree of change in their muscle synergies as reflected by their similar across-session scalar-product values (p>0.05; Fig. S2B). After the synergies of each session from all subjects of each group were *k*-means clustered (see Fig. S3A-B for the *k*-means silhouette values), a comparison of the session-1 cluster centroids with the session-3 centroids in both groups revealed no obvious difference in

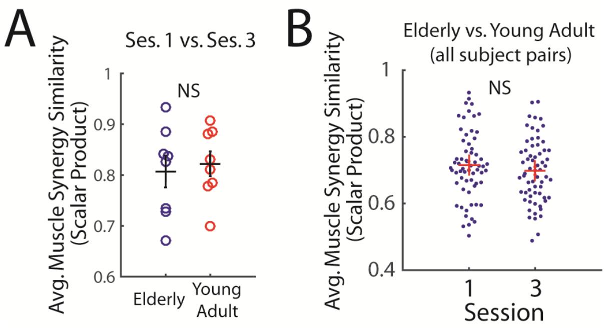

Fig. S2. Standard muscle synergy similarity analysis. *A*, In each age group, the session-1 and session-3 synergies of each subject were matched by maximum scalar product (SP). The average SP for elderlies did not significantly differ (NS) from that for young adults (t-test, p > 0.05, mean ± SE). *B,* In each session, the synergies of every elderly-young adult subject pair were matched. The average SP for session 1 did not significantly differ (NS) from that for session 3 (t-test, p > 0.05, mean ± SE).

how the synergies changed from session 1 to 3 between the two age groups (Fig. S3C-D). Indeed, even the session-1-specific clusters in both groups involved similar muscles, including deltoid (medial and posterior parts) and biceps brachii (Biceps) (Fig. S3C-D). The similarity between the elderly and young-adult synergies persisted when synergies were extracted from the combined EMGs of sessions 1 and 3 (Fig. S4). Thus, conventional muscle synergy analysis did not reveal any salient age-related difference in the muscle synergies of either session, and how the synergies are changed by learning.

II. Materials and Methods

A. Subjects

Two groups of 8 male right-handed subjects each participated in this study: the elderly group (age range of 68 to 75 years old, mean age of 71.1 ± 2.4 [SD]), and the young adult group (age range of 22 to 26, mean age of 23.8 ± 1.7). By filling a questionnaire, all subjects verified, to the best of their knowledge, that they had no visual impairment and any medical condition that would influence their motor abilities, and had never received any regular bowling-related training prior to the study. The study's protocol was reviewed and approved by the Joint Chinese University of Hong Kong – New Territories East Cluster Clinical Research Ethics Committee (protocol

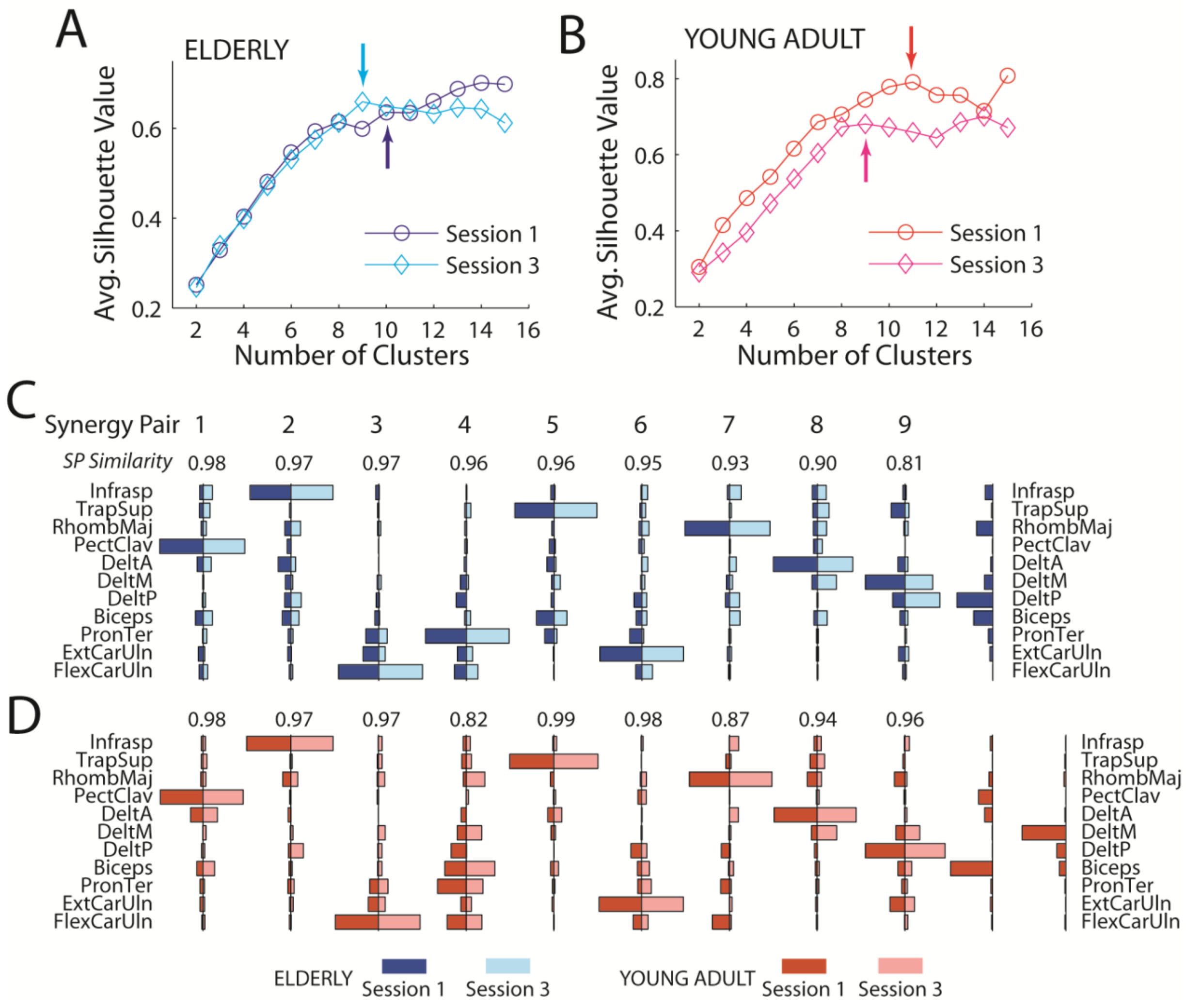

Fig. S3. Conventional muscle synergy analysis performed on the bowling EMGs. For each session, *W*'s from all subjects of the group were pooled and *k*-means clustered. *A,B,* The number of clusters was determined by finding the number at which the mean silhouette value plateaued (arrows indicating optimal number). In *A*, for session 1 the mean silhouette peaked at 14 clusters, but partition at this number was obviously too fragmented to be heuristically useful. Hence, we decided to select the first local peak (10 clusters). *C,D,* The cluster centroids from the 2 sessions were matched by maximum scalar product (SP).

2016.203), and all subjects provided written informed consent before experimentation.

B. *Virtual bowling game*

All subjects were trained to play, with their non-dominant left arm, a virtual reward-based bowling game constructed using a Kinect sensor (Microsoft Corp., Redmond, WA, USA), and a video game console (Xbox One; Microsoft, Redmond, WA, USA) connected to a widescreen liquid-crystal monitor (1080p). We purposefully assigned the bowling arm to the non-dominant side to make the game more difficult to learn, and thus amplify the effects of training on the muscle synergies. The game environment was provided by Kinect Sports Rivals (Rare; Twycross, UK), which contains a readily usable environment for 10-pin bowling. The Kinect sensor, always placed at 89 cm above ground and 80 cm in front of the monitor, was calibrated before every game session to correctly track the body movement of each subject. A warning was given on the display when the sensor failed to capture the subject's whole-body motion during the game because of dark clothing items or nearby obstructions.

Just like the ordinary bowling games, the explicit goal for the subject was to knock over as many pins at the lane's end as possible with the virtual ball. To start a trial, the subject first stood in front of the Kinect sensor, picked up a virtual ball using the left hand, and held it by making a fist. The subject was then instructed to line his hand up to the center triangle on the virtual bowling lane with the help of the Kinect sensor, which would then send the ball straight to the lane's center. Then, an arm swing motion picked up by the sensor served to simulate the throwing of the ball whose virtual release was signified by the opening of the non-dominant hand together with an accelerated wrist flexion during mid-swing. After release, the ball would then run virtually along the lane on the display.

The exact kinematic determination of the number of knocked-down pins in our Kinect game has not been published

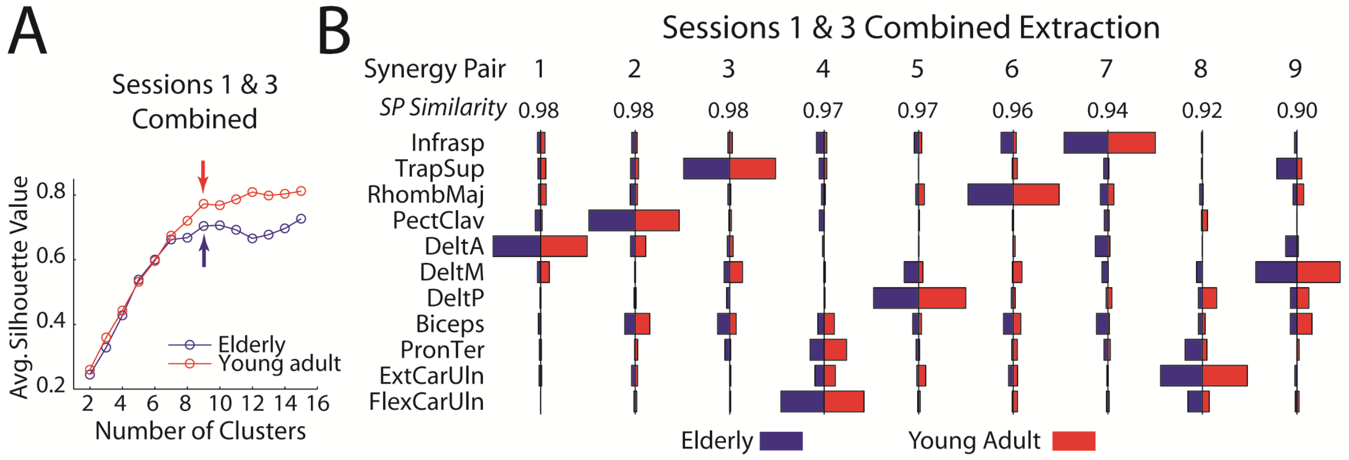

Fig. S4. Conventional muscle synergy analysis performed on the combined session-1 and -3 EMGs. Muscle synergies were extracted from the combined EMGs. Synergies from all subjects were pooled together and *k*-means clustered. *A*, The number of clusters for each age group was determined by finding the number at which the mean silhouette value plateaued (arrows indicating optimal numbers). *B*, Cluster centroids from the two age groups were matched by maximum scalar product. Note that the centroids shown here were used to initialize NMF to extract session-fixed *W* in the first stage of variability analysis.

or made available by the game company. But according to prior studies on the biomechanics of real ordinary bowling, the number of knocked-down pins would depend on the ball's direction and speed [4], which in turn would likely depend on the speed of shoulder and elbow flexion [5], and to the timing [4] and speed of wrist flexion and internal rotation [5]. These biomechanical determinants of high scores appear consistent with the experiences of the players of our virtual bowling game reported in online gaming forums [6]. Importantly, since the ball's trajectory would be affected by the position of the subject's body relative to the lane's center [7], in each trial the subject was instructed to always stand at the same position in front of the sensor marked on the floor, so that the distance between toe on the right side and the Kinect sensor was fixed at 208 cm. Since it is natural for a bowler to control the ball's direction by adjusting the position and direction of the foot contralateral to the bowling arm [8], by fixing the right-toe-to-sensor distance the subject would be forced to knock down more pins by adjusting only shoulder and wrist motions. No explicit instruction was otherwise given to the subject regarding the ball speed and direction required for knocking down pins.

Each subject was trained alone on the bowling game for 3 sessions held on separate, consecutive days. In every session, the subject was required to bowl for ~30 trials (26.85 ± 4.11 trials). Each trial consisted of 2 throws. If the first throw was not a strike (i.e., all 10 pins down), with the second throw the subject could aim to knock down the remaining pins. All behavioral, myoelectric and kinematic data (see below) were only collected from the first throw of each trial, however, so that the data of all trials should reflect the subjects' aim to score from the initial 10 pins. After each throw, performance was fed back to the subject through a direct visual display of the stricken-down and remaining pins, and a verbal and visual report of the number of knocked-down pins from the game system.

C. Measures of performance

We quantified the across-session bowling performance of every subject with two different measures. The first measure used was simply the average number of pins knocked down (a score out of 10) across all first throws of a session, one that the subject was *explicitly* aiming to maximize during the game. The second measure used was the release speed of the virtual ball, which should correlate directly with the maximum speed of wrist flexion attained during the throw. We estimated this speed by calculating the peak forward speed of a reflective marker placed on the left wrist (see below). Even though the ball release speed was presumed to contribute, together with other factors such as the ball trajectory, to the number of knocked-down pins, we did not observe any statistically significant correlation between the average ball release speed and the average bowling score across subjects in either group (elderly, Pearson's r = 0.32, p = 0.22; young adult, r = 0.19, p = 0.47) or in both groups (r = 0.26, p = 0.15). Thus, the ball release speed captures an aspect of kinematic changes that is at best only partially related to the explicit goal of the game.

D. Kinematics and EMG recordings

Kinematics and multi-channel electromyographic data (EMGs) were collected from each subject during all bowling trials in sessions 1 and 3. For kinematics, since the release speed of the virtual ball was presumably related to the velocity of wrist flexion (see above), we estimated the peak wrist flexion velocity by tracking the peak forward speed of the forearm endpoint. This was accomplished by monitoring the 3D position of a reflective marker on the left radial styloid process (marker LWRA in Vicon's documentation) [9]. Its position during each trial was tracked and recorded, at 100 Hz, by 10 high-resolution infrared cameras (Vantage V5, Vicon; Centennial, CO, USA). The maximum speed attained by the marker in the forward (i.e., wrist flexion) direction was calculated in the Nexus software environment (Vicon, Centennial, CO, USA).

For muscle activities, surface EMGs were recorded digitally from 11 upper-limb and back muscles on the non-dominant side, including infraspinatus (Infrasp); trapezius, upper part (TrapSup); rhomboid major and/or trapezius, lower part (RhombMaj); pectoralis major, clavicular head (PectClav); deltoid, anterior (DeltA), medial (DeltM) and posterior (DeltP)

parts; biceps brachii (Biceps); pronator teres (PronTer); extensor carpi ulnaris (ExtCarUln); and flexor carpi ulnaris (FlexCarUln). All EMG electrodes were placed in accordance to the guidelines of the Surface Electromyography for the Non-Invasive Assessment of Muscles – European Community Project (SENIAM; www.seniam.org) to the extent possible, and were attached to skin surface using double-sided tape and self-adherent bandage wrap (3M Coban^{TM}). All EMG signals were recorded using a wireless EMG system (Trigno, Delsys; Natick, MA, USA) at 2 kHz. Importantly, in both sessions 1 and 3, before the bowling trials, for every muscle the EMG elicited during maximum voluntary contraction (MVC) was recorded.

E. EMG preprocessing and normalization

Digital EMG data from both the MVC and bowling trials were read from the source data files (c3d format) using the open-source Biomechanical Toolkit (http://biomechanical-toolkit.github.io/). All EMG signals were high-pass filtered at 50 Hz (finite impulse response filter, or FIR; 50th order), rectified, and low-pass filtered at 20 Hz (FIR; 50th order) using custom Matlab routines [10]. Next, the EMGs of each muscle were time-averaged in 25-ms time bins [10]. For each session of each subject, the pre-processed bowling EMGs of each muscle were then normalized to either the maximum value recorded in the MVC trials or the maximum recorded in the bowling trials, whichever was higher. Note that the EMGs of each muscle were *not* normalized to unit variance, as in many previous studies [11,12], because the variance of synergy activations is itself a parameter of interest in this study (see below). No time normalization was performed on the EMGs.

F. Conventional muscle synergy analysis

To have a first assessment of how the upper-limb muscle synergies may differ between groups and sessions, we performed a standard muscle synergy analysis on the EMGs of each subject from each session. As in many previous studies (e.g., [10-16]), multi-muscle EMGs were modeled to be generated by the linear combination of time-invariant muscle synergies,

$$D = WC + \varepsilon\,, \qquad (1)$$

where D is the EMG time series for the recorded muscles (as row vectors), W is the matrix comprising the synergies (as column vectors), C is the matrix denoting the time series of activation coefficients for the synergies (as row vectors), and ε is the residual unexplained by the model. The W and C matrices were identified from the preprocessed and normalized EMGs using the standard non-negative matrix factorization algorithm (NMF) [1] that assumes Gaussian noise in the data [2].

To determine the number of muscle synergies needed for data reconstruction (i.e., the EMG dimensionality), NMF was applied to successively extract 1, 2, …, 11 synergies from the EMGs of each session. The number of muscle synergies deemed adequate was determined to be the minimum number required for an EMG-reconstruction R^2 of ≥90%. The 90% threshold here differs from the usual 80% threshold used in previous studies (e.g., [12]). Since the EMG here was normalized to the muscle's MVC magnitude instead of to unit variance, a higher threshold was necessary to capture the structure embedded in the muscles with lower magnitudes in the bowling trials after data normalization. To prevent the extracted W and C from representing a suboptimal solution at a local minimum on the error (ε) surface, each extraction run was repeated 20 times, each time with W and C initiated with different uniformly distributed random values between 0 and the maximum EMG value. The solution yielding the highest R^2 was selected for downstream analysis. For every extraction run, the NMF update rules were terminated when a between-iteration change of EMG-reconstruction R^2 <0.001% was observed in 20 consecutive iterations [10]. Every muscle synergy was normalized to unit vector, and the synergy's activation multiplied by the original synergy-vector magnitude, so that all C's represented activation drives of unit vectors.

To facilitate between-group and between-session comparisons of the muscle synergies, for each of the elderly and young adult groups the synergies identified from all subjects in each session were classified into clusters by *k*-means implemented on Matlab (kmeans.m, options of squared Euclidean distance and 5,000 replicates). Clustering was also implemented on the pooled session-1 and session-3 synergies of each age group. For every *k*-means run, clustering was performed with 2, 3, … 15 clusters; the optimal number of clusters was determined by finding the number of clusters at which the average silhouette value plateaued [17]. The cluster centroids were normalized to unit vectors for downstream analyses.

For every subject, the magnitude-normalized synergies for session 1 were matched to those for session 3 by maximizing the total scalar product values (SP) between the synergy pairs [3]. The overall similarity between the two synergy sets was thus quantified by the average SP across the synergies. SP's were also computed between the synergy sets of each session of every elderly-young-adult subject pair (8 x 8 = 64 pairs), and for each subject group, between the synergy cluster centroids of session 1 and those of session 3.

G. Correlating variability of muscle-synergy activations with performance measures

In this study, we ask whether the initial (i.e., session 1) and change (i.e., from session 1 to 3) of across-trial variability of W and/or C may correlate with the change in bowling performance from session 1 to 3, in both elderlies and young adults. In our first stage of variability analysis, we focused on finding statistically significant correlations with covariates that denote the initial or change in C variability in either activation magnitude or activation timing, assuming that W is fixed across all trials of a session, and that the numbers of synergies for both sessions for all subjects are the same (= 9; see below). In this regard, after identifying W and C of each session (the procedure for identifying them will be described below), we quantified the C-magnitude variability of every synergy by calculating the variance of the trial-maximum magnitude of C (C_{max}), across all trials of each session. We then proceeded to examine whether the following correlations were statistically significant across all elderly or young-adult subjects:

(1) The average (across each subject's synergies) initial (session 1) C_{max} variance *vs.* the change in bowling score or change in ball release speed;

(2) The maximum (across each subject's synergies) initial C_{max} variance *vs.* the change in bowling score or change in ball release speed;
(3) The average (across each subject's synergies) change (from session 1 to 3) of C_{max} variance *vs.* the change in bowling score or change in ball release speed;
(4) The maximum (across each subject's synergies) change of C_{max} variance *vs.* the change in bowling score or change in ball release speed.

Thus, for each subject group, a total of 4 (covariates) x 2 (bowling score or ball speed) = 8 correlations were performed. The strength and statistical significance of all correlations were quantified by the Pearson's correlation coefficient (*r*).

In addition, we correlated the variability of activation timing of *C* with the change in performance measures. For every synergy, the time point within the trial at which C_{max} occurred (T_{Cmax}) was noted. Timing variability was quantified by the variance of the time interval between the T_{Cmax}'s of any 2 synergies within the synergy set. Thus, for 9 synergies, there were $^9C_2$ = 36 time-interval variance values from each subject. As in the *C*-magnitude variability analysis, for every of the 36 time-interval variances, both the initial (session 1) and the change (from session 1 to 3) of variance were correlated with either the change in bowling score or ball release speed. Thus, a total of 36 (time-interval variances) x 2 (initial or change of variance) x 2 (bowling score or ball speed) = 144 correlations were performed for the elderly and young-adult groups, respectively.

The above-described *C*-variability analysis assumes a *W* that is fixed within a session. Since our synergy clustering analysis indicated that the *W*'s for both sessions for both subject groups could be grouped into 9 clusters (Fig. S4), for this *C*-variability analysis we enforced 9 muscle synergies to be extracted from the EMGs of each session of each subject. Also, as we studied the *change* of *C* variability of a synergy from session 1 to 3, there was the necessary assumption that the *C*'s of both sessions represented the activations of the same coordinative entity, *W*, from session 1 to 3, even though this session-fixed *W* may itself be modified across sessions. To identify the session-1 and session-3 *W*'s so that the synergy vectors in the former would suitably correspond to those of the latter, for both sessions we initialized the NMF update rule for *W* with the same matrix. This initial matrix we used was composed of the 9 *W*-cluster centroids derived by applying *k*-means to the *W*'s extracted from the combined EMGs of *both* sessions 1 and 3, and collected from all subjects of either group. This way, when extracting the subject- and session-specific *W*, the NMF was armed with prior knowledge of a generalized *W* based on its average representation across all subjects and sessions; extraction of *W* would therefore amount to suitably updating this "*W* prior" to accommodate any data features peculiar to the session and subject concerned (see below).

H. Modeling EMG variability by trial-specific muscle synergies

In the first stage of analysis described above, we modeled the across-trial variability of EMGs to originate from the spatiotemporal variability of *C* that activates a session-fixed *W*. In the second stage of analysis, we further explored a model that allows both *W* and *C* to be variable across trials, and examined whether the initial and change of *W* or *C* variability may correlate with the change of performance measures across subjects. In conventional muscle synergy analysis, *W* is always regarded as an invariant structure that accounts for EMG variability. To identify *W*, NMF or another algorithm is used to discover features embedded within the EMG variability. Here, on the other hand, we intend to examine muscle synergy as an entity that itself exhibits trial-to-trial variability. A new computational procedure is needed to identify this variation of *W* from trial-specific muscle synergies. Importantly, the averages of trial-specific synergies across trials should still *globally* explain the data by spanning most of the EMG subspace, so that a trial-specific *W* should represent a variant of a "true global *W*" rather than just a fit to the data peculiarities and noise of the trial.

To accomplish our computational goals, we exploited the fact that the NMF update rules are iterative steps for estimating the *W* and *C* by maximizing the likelihood of observing the EMGs given any initial estimates of *W* and *C*, which correspond to prior knowledge of how the muscle synergies and their activations should be structured [2,3,18]. It follows that trial-specific *W* and *C* can be suitably estimated from the EMG of each trial by initiating the NMF with the global *W* and *C* extracted from the entire EMG data set comprising all trials, which should represent *a priori* knowledge of what the trial-specific *W* and *C* should be close to. To identify trial-specific muscle synergies, we implemented, for every subject and session, the following steps:

(1) We applied NMF to EMGs of all trials of both sessions with randomized *W* and *C* as initial estimates, as described above, and extracted the global *W* and *C* with specifications identical to those used in conventional muscle synergy analysis (see above).

(2) For every trial, we applied NMF again to the EMGs of that trial, this time initiating the algorithm with the global *W* and *C* from (1), so that the "*W* prior" and "*C* prior" were updated by the algorithm to account for any trial-specific EMG variations.

If the above steps did return trial-specific *W*'s that reflect genuine muscle-synergy fluctuations across trials given the *W* prior (instead of just any *W*'s that maximize the EMG variance explained), in each trial, every trial-specific synergy should be reasonably similar to its own *W* prior from whose updating it was derived by NMF. We evaluated to what extent this was the case by matching every set of trial-specific *W*'s to the *W* prior synergies that initialized the NMF by maximizing the total scalar-product values (see above). For every *W*-prior synergy, we then calculated the percentage of trials whose trial-specific synergies derived from the same *W*-prior synergy were matched

back to the W-prior synergy itself. A high percentage for all W-prior synergies would indicate that the NMF, when applied to the trial EMG with the W and C prior, did not return drastically different synergies.

In some trials, we noted that a W-prior synergy was updated by NMF to result into a trial-specific synergy that was best matched to another W-prior synergy (for instance, prior synergies 1 and 2, after NMF updating, became trial-specific synergies closest to prior 2 and 1, respectively). If this happened, we reassigned the trial-specific synergies to their closest prior synergies, so that subsequent calculations of the W variability would be based on the variance of a group of trial-specific synergies that were all closest to the same prior. We found that this reassignment step did increase the Pearson's r slightly when W variability was correlated with behavioral performance measures, but did not result in any qualitative change in study's main conclusions.

For each session of every subject, W variability (WV) was quantified by the total across-trial variance of the trial-specific synergies:

$$WV = \sum_{i=1}^{11} Var([W_{i1}, W_{i2} \ldots, W_{iN}]) \,, \qquad (2)$$

where N is the number of trials, and i is an index for the 11 muscles. All trial-specific W's were normalized to unit vectors before WV calculations. As in the C_{max} variability analysis described above, both the initial (session 1) and change (from session 1 to 3) of mean and maximum WV (across the synergies of a subject) were correlated with the change in average bowling score and ball release speed, across either the elderly or young adult subjects. Variability and correlation analyses for C_{max} – this time derived from C's that activate trial-specific W's – were likewise conducted in steps identical to those described above for C's that activate session-fixed W's.

III. Tables

TABLE S1. Correlating variability of C activation magnitude with performance measures (with session-fixed W)

Pearson's r values for all correlations are listed. Their corresponding p values are shown in parentheses. Statistically significant values are shown in bold.

**ELDERLY**		
Predictor	Response variable	
	Ses.1-3 Change of Average Score	Ses.1-3 Change of Ball Speed
Mean Ses. 1 C_{max} Var.	0.20 (0.63)	***-0.74 (0.03)***
Max. Ses. 1 C_{max} Var.	0.32 (0.44)	-0.61 (0.11)
Mean Ses. 1-3 Change of C_{max} Var.	0.03 (0.95)	0.43 (0.29)
Max. Ses. 1-3 Change of C_{max} Var.	-0.05 (0.91)	0.01 (0.97)
**YOUNG ADULT**		
Predictor	Response variable	
	Ses.1-3 Change of Average Score	Ses.1-3 Change of Ball Speed
Mean Ses. 1 C_{max} Var.	0.01 (0.98)	-0.17 (0.69)
Max. Ses. 1 C_{max} Var.	0.02 (0.96)	-0.14 (0.74)
Mean Ses. 1-3 Change of C_{max} Var.	0.48 (0.23)	-0.03 (0.94)
Max. Ses. 1-3 Change of C_{max} Var.	***0.93 (0.0007)***	-0.07 (0.87)

TABLE S2. Correlating variability of W with performance measures (with trial-variable W and C)

Pearson's r values for all correlations are listed. Their corresponding p values are shown in parentheses. Statistically significant values are shown in bold.

**ELDERLY**		
Predictor	Response variable	
	Ses.1-3 Change of Average Score	Ses.1-3 Change of Ball Speed
Mean Ses. 1 W Total Var.	0.50 (0.20)	0.31 (0.46)
Max. Ses. 1 W Total Var.	0.17 (0.69)	0.13 (0.76)
Mean Ses. 1-3 Change of W Total Var.	-0.23 (0.59)	-0.09 (0.84)
Max. Ses. 1-3 Change of W Total Var.	0.14 (0.74)	-0.34 (0.41)
**YOUNG ADULT**		
Predictor	Response variable	
	Ses.1-3 Change of Average Score	Ses.1-3 Change of Ball Speed
Mean Ses. 1 W Total Var.	-0.59 (0.13)	0.11 (0.80)
Max. Ses. 1 W Total Var.	-0.54 (0.16)	0.07 (0.88)
Mean Ses. 1-3 Change of W Total Var.	***0.78 (0.02)***	-0.25 (0.56)
Max. Ses. 1-3 Change of W Total Var.	***0.80 (0.02)***	-0.51 (0.19)

TABLE S3. Correlating variability of C activation magnitude with performance measures (with trial-variable W and C)

Pearson's r values for all correlations are listed. Their corresponding p values are shown in parentheses. Statistically significant values are shown in bold.

**ELDERLY**		
Predictor	Response variable	
	Ses.1-3 Change of Average Score	Ses.1-3 Change of Ball Speed
Mean Ses. 1 C_{max} Var.	-0.08 (0.85)	-0.65 (0.08)
Max. Ses. 1 C_{max} Var.	0.10 (0.81)	-0.09 (0.83)
Mean Ses. 1-3 Change of C_{max} Var.	-0.04 (0.93)	0.59 (0.12)
Max. Ses. 1-3 Change of C_{max} Var.	-0.29 (0.48)	0.15 (0.72)
**YOUNG ADULT**		
Predictor	Response variable	
	Ses.1-3 Change of Average Score	Ses.1-3 Change of Ball Speed
Mean Ses. 1 C_{max} Var.	-0.21 (0.61)	0.26 (0.51)
Max. Ses. 1 C_{max} Var.	-0.40 (0.33)	0.50 (0.21)
Mean Ses. 1-3 Change of C_{max} Var.	***0.77 (0.03)***	-0.39 (0.34)
Max. Ses. 1-3 Change of C_{max} Var.	0.34 (0.41)	-0.34 (0.41)

IV. References

[1] D. D. Lee, H. S. Seung, "Learning the parts of objects by non-negative matrix factorization," *Nature*, vol. 401, no. 6755, p. 788, Oct. 1999.

[2] K. Devarajan, V. C. K. Cheung, "On nonnegative matrix factorization algorithms for signal-dependent noise with application to electromyography data," Neural Compt., vol. 26, no. 6, pp. 1128-68, Jun. 2014.

[3] V. C. K. Cheung et al., "Central and sensory contributions to the activation and organization of muscle synergies during natural motor behaviors," *J. Neurosci.*, vol. 25, no. 27, pp. 6419-34, Jul. 2005.

[4] K. Kimura et al., "Measurement and analysis of bowling swing motion using 3D acceleration and gyro sensors," in: *SICE Annual Conference 2011*, IEEE, Sep. 2011, pp. 1115-1119.

[5] R. Razman et al., “Anthropometric and strength characteristics of tenpin bowlers with different playing abilities,” Biol. Sport, vol. 29, no. 1, pp. 33-38, Mar. 2012.

[6] D. T. G. Reviews (2014, May), Kinect Sports Rivals: tips to do well in the bowling game [online]. Available: https://www.dtgre.com/2014/05/kinect-sports-rivals-tips-to-do-well-in.html?m=1

[7] R. Razman et al., “Front foot slide variability and its relation to tenpin bowling performance,” in: *ISBS-Conference Proceedings Archive* (Vol. 1 No. 1), 2010.

[8] P. D. Hurrion et al., “Simultaneous measurement of back and front foot ground reaction forces during the same delivery stride of the fast-medium bowler,” *J. Sports Sci*., vol. 18, no. 12, pp. 993-7, Jan. 2000.

[9] K. J. Middleton et al., “The influence of elbow joint kinematics on wrist speed in cricket fast bowling,” *J. Sports Sci*., vol. 33, no. 15, pp. 1622-31, Sep. 2015.

[10] V. C. K. Cheung et al., “Stability of muscle synergies for voluntary actions after cortical stroke in humans,” *PNAS*, vol. 106, no. 46, pp. 19563-8, Nov. 2009.

[11] G. Torres-Oviedo, L. H. Ting, “Subject-specific muscle synergies in human balance control are consistent across different biomechanical contexts,” *J. Neurophysiol*., vol. 103, no. 6, pp. 3084-98, Apr. 2010.

[12] V. C. K. Cheung et al., “Muscle synergy patterns as physiological markers of motor cortical damage,” *PNAS*, vol. 109, no.36, pp. 14652-6, Sep. 2012.

[13] M. C. Tresch et al., “The construction of movement by the spinal cord,” *Nat. Neurosci*., vol. 2, no. 2, p. 162, Feb. 1999.

[14] P. Saltiel et al., “Muscle synergies encoded within the spinal cord: evidence from focal intraspinal NMDA iontophoresis in the frog,” *J. Neurophysiol*., vol. 85, no. 2, pp. 605-19, Feb. 2001.

[15] T. Takei et al., “Neural basis for hand muscle synergies in the primate spinal cord,” *PNAS*, vol. 114, no. 32, pp. 8643-8, Aug. 2017.

[16] Q. Yang et al., “Motor primitives are determined in early development and are then robustly conserved into adulthood,” *PNAS*, vol. 116, no. 24, pp. 12025-34, Jun. 2019.

[17] P. J. Rousseeuw, “Silhouettes: a graphical aid to the interpretation and validation of cluster analysis,” *J. Comput. Appl. Math*., vol. 20, pp.53-65, Nov. 1987.

[18] V. C. K. Cheung, M. C. Tresch, “Non-negative matrix factorization algorithms modeling noise distributions within the exponential family,” in *2005 IEEE Engineering in Medicine and Biology 27th Annual Conference*, IEEE, Jan. 2006, pp. 4990-4993.
